# HED, a Human-Engineered Domain, Confers a Unique Fc-Binding Activity to Produce a New Class of Humanized Antibody-like Molecules

**DOI:** 10.3390/ijms24076477

**Published:** 2023-03-30

**Authors:** Zhiqiang Zhu, Peeyush N. Goel, Cai Zheng, Yasuhiro Nagai, Lian Lam, Arabinda Samanta, Meiqing Ji, Hongtao Zhang, Mark I. Greene

**Affiliations:** 1 Department of Pathology and Lab Medicine, Perelman School of Medicine, University of Pennsylvania, Philadelphia, PA 19104, USA; 2 Children’s Hospital of Philadelphia (CHOP), Philadelphia, PA 19104, USA

**Keywords:** engineered antibody, humanized, HED bodies, breast cancer, IFN-γ

## Abstract

Our laboratory has identified and developed a unique human-engineered domain (HED) structure that was obtained from the human Alpha-2-macroglobulin receptor-associated protein based on the three-dimensional structure of the Z-domain derived from Staphylococcal protein A. This HED retains µM binding activity to the human IgG1CH2-CH3 elbow region. We determined the crystal structure of HED in association with IgG1’s Fc. This demonstrated that HED preserves the same three-bundle helix structure and Fc-interacting residues as the Z domain. HED was fused to the single chain variable fragment (scFv) of mAb 4D5 to produce an antibody-like protein capable of interacting with the p185Her2/neu ectodomain and the Fc of IgG. When further fused with murine IFN-γ (mIFN-γ) at the carboxy terminus, the novel species exhibited antitumor efficacy in vivo in a mouse model of human breast cancer. The HED is a novel platform for the therapeutic utilization of engineered proteins to alleviate human disease.

## 1. Introduction

Staphylococcal protein A (SpA) is an important virulence factor found on the surface of Staphylococcus aureus. The protein has five tandem Z-domains (E-D-A-B-C) at the N-terminus and a cell wall anchoring domain at the C-terminus. These domains are responsible for interacting with known proteins, such as tumor necrosis factor receptor 1 (TNFR1) [1] and von Willebrand factor (vWf) [2]. The constant region (Fc) of IgG [3] and the Fab region of VH3 immunoglobulin [4] interact with SpA to bind human or animal immunoglobulin through the Z-domains. It has been proposed that Fc interactions with SpA shield staphylococci from opsonic phagocytic killing [5]. Additionally, SpA cross-links the VH3 type B cell receptor (surface IgM) resulting in proliferative clonal expansion as well as apoptosis of certain activated host B cells [6]. Resolution of the complex structure of the SpA B-domain and the Fc fragment of human IgG1 (hIgG1Fc) [3] (PDB Code: 1fc2) has revealed that the Z-domain binds to the elbow region between CH2 and CH3 domains of Fc. An accessible surface of helix1 and helix2 of the B-domain forms the interface with Fc. Contrarily, SpA uses a distinct surface derived from helix2 and helix3 to interact with IgM Fab regions, as deduced from the complex structure of the SpA D-domain and the Fab fragment of a human IgM antibody [7] (PDB code: 1dee).

Graille et al. investigated a ternary complex containing Fc, Z-domain, and Fab molecules. The model indicated that a single Z domain may simultaneously bind Fab and Fc [7]. There have been reports of crystal structures of individual domains, and higher resolution structures of a single C domain and two tandem B domains connected by a conserved linker can exhibit extensive multi-scale conformational heterogeneity [8]. This structural characteristic may provide structural flexibility to SpA, allowing it to bind to numerous partners. The Z-domain has distinctive biophysical properties, solvent accessibility, and unexpected stability characteristics that restrict its proteolysis [9]. It lacks cysteine residues, making it a valuable structural platform for biomedical engineering. Previously our laboratory employed the Z helical domain derived from the SpA B-domain to fuse with the single chain variable fragment of humanized mAb 4D5 (4D5scFv) [10]. This species of protein could recruit circulating human IgGs and attracted immune cells carrying Fc receptors to interact with p185Her2/neu expressing cells targeted by 4D5scFv. These antibody-like ZZ proteins were observed to have potent complement-dependent cytotoxic (CDC) activity on T6-17 cells and antibody-dependent cell-mediated cytotoxicity (ADCC) activity on BT474 cells. 4D5scFvZZ was more effective than the scFv of mAb4D5 at inhibiting the development of tumors in vivo [10].

SpA Z-domains have been fused with proteins to boost their immunogenicity in vivo. When antigen-presenting cells (APCs) have surface Igs, ZZ-antigen conjugates exhibit increased presentation to an Ag-specific T-cell hybridoma [11] The binding of ZZ to Igs on the surface of APC cells is an efficient strategy for peptide-based vaccines to promote immunity [11,12]. When employed as an immunogen in mice, purified recombinant SpAKKAA (the nontoxigenic mutant of SpA) induces B cell responses to the virulence antigen protein A [12]. Consequently, monoclonal antibodies 5A10, 3F6, and 3D11 that target separate regions of SpA have been developed. The antibodies 5A10 and 3F6 identify SpA Z-domains, whereas 3D11 binds SpA domains A, B, and C [13]. The potential immunogenicity of the Z-domain in humans hinders the clinical use of the Z-domain or other fusion proteins. To reduce the immunological reactions and adverse effects of the Z-domain, we generated a structurally comparable human protein with Fc-binding ability into a human-engineered domain (HED). The HED retains the domain Z residues that bind with Fc.

We solved the crystal structure of HED in complex with the Fc fragment of human IgG1(hIgG1Fc). HED was subsequently fused with 4D5scFv to generate an antibody-like species that could bind HER2-positive tumor cells through its single chain Fv portion and Ig antibodies with µM affinity via its humanized Z-domain. Additionally, the humanized antibody-like protein was fused with mIFN-γ to generate a new class of proteins known as HED-bodies. The HED-body displays potent ADCC activity and tumor growth suppression in vivo.

## 2. Results

### 2.1. Model Design of HED

As a fundamental structural component of Staphylococcus Protein A, the Z domain possesses a three-helix bundle scaffold, as characterized by NMR structures of the B-domain and its mutant forms (such as 1BDC, 1SS1, in PDB) [14,15]. The crystal structures of various Z-domains reflect the important stability properties of the main chain’s atoms. We selected the domain D in the complex structure of the SpA domain D and IgM Fab (PDB code 1DEE) as a model to search for comparable three-dimensional structures in the Protein Data Bank using the DALI webserver [16].

A total of 430 structures with a Z-score greater than 2.0 were identified. As anticipated, the majority were Z-domains derived from bacteria or Z-domain-like affibodies. As our objective was to reduce immunogenicity when using therapeutic proteins, we considered the human origin of the proteins as a limiting factor in our structure search. We deemed it critical that the discovered protein has solvent-exposed surfaces to promote independent folding and prevent aggregation under physiological settings, among other structural characteristics. In addition, an optimal structure would possess “no” cysteine residues and display minimum gaps of primary sequence to facilitate its expression and maintain its essential structure.

**Figure 1:**
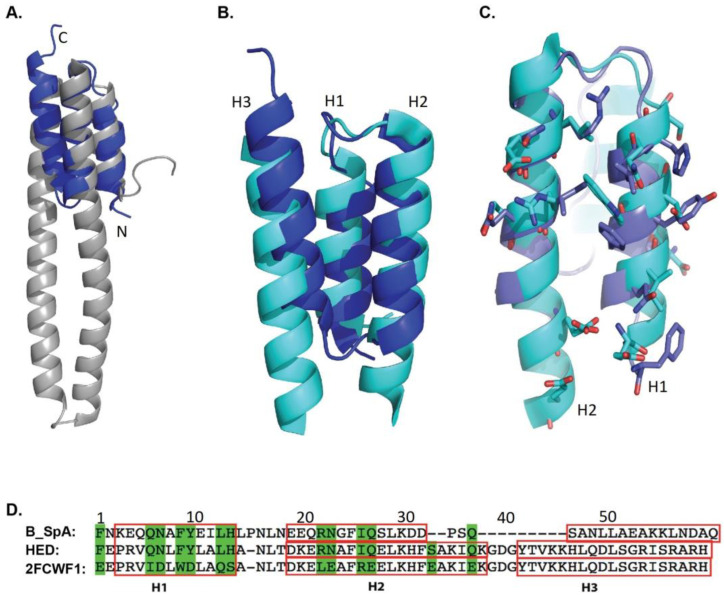
Design of HED domain. (**A**) Three-dimensional structural alignment of a human Alpha-2-macroglobulin receptor-associated protein (Alpha-2-MRAP, Chain A of PDB 2FCW, grey) with the domain D (blue) of Staphylococcus Protein A in the complex crystal structure with the Fab fragment of a human IgM antibody (PDB code: 1DEE). (**B**) The corresponding region of Alpha-2-MRAP to the D-domain of SpA (2FCWF1) was selected for modeling and colored in cyan. (**C**) The original model of 2FCWF1 was superimposed on the structure of the B-domain of SpA from its complex with the Fc fragment of human IgG1 (PDB code: 1FC2). The interface residues of the B-domain and the Fc fragment and the corresponding residues in the model of 2FCWF1 are shown as sticks on their cartoon model, and their carbon atoms are painted in blue and cyan, respectively. (**D**) The primary sequence alignment of 2FCWF1, B-domain of SpA, and HED domain. The residues mutated from Alpha-2-MRAP to the B-domain of SpA are shown in green shadow, and the helixes of their secondary structure and the predicted helixes of HED are labeled as H1, H2, and H3 and highlighted with red boxes.

With these imposed search criteria, the structure model of the human alpha-2-macroglobulin receptor-associated protein (Alpha-2-MRAP, chain A of PDB: 2FCW) was identified and superimposed onto the domain D structure [17]. The Z-score and the RMSD of the main chain are 2.7 and 2.2 angstroms. The structure alignment of hA2MRAP (chain A of 2FCW) and domain Z (chain G of 1DEE) is shown in Figure 1A. Only part of the RAP could be aligned to the Z-domain as there is a long fragment that is used for binding the low-density lipid receptor (LDLR) that extends between the second and the third helix in RAP. Therefore, we removed the LDLR binding fragment and linked the second and the third helix using a short linker: Gly-Asp-Gly (Figure 1B). A new polypeptide with a continuous sequence was created (2FCWF1 sequence in Figure 1D).

Employing the shortened sequence from 2FCWF1 (Figure 1D), we built a new three-dimensional structural model using the webserver of SWISS-MODEL [18] (https://swissmodel.expasy.org (accessed on 5 February 2023)). We then aligned the model to the SpA B-domain from the complex structure of the B-domain with the Fc fragment of human IgG1 (Figure 1C). The residues in the 2FCWF1 model that correspond to Fc-interacting residues in the B-domain are shown in green in Figure 1D. These residues (1, 6, 7, 9, 10, 13, 14, 22, 23, 26, and 27 of 2FCWF1) were then substituted with the residues of the B-domain to generate a new sequence to obtain a humanized Z-domain (HED, Figure 1D). The immunogenicity of the SpA domain and HED were determined using a computational model [19,20,21] and, as predicted by the model, both SpA and HED are not very immunogenic (Figure S1A,B).

### 2.2. Crystal Structure of HED Complexed with hIgG1Fc

To determine if the “humanized” Z-domain interacts with the Fc fragment of human IgG1, a plasmid was constructed that expresses a fusion protein of glutathione-S-transferase (GST) and HED with a TEV protease site. We were able to co-express the GST-HED fusion protein with hIgG1Fc in bacteria BL21 (DE3). When we purified the GST-HED fusion protein using Glutathione Sepharose 4B, hIgG1Fc was also co-purified (Figure S2A). Cleavage of the TEV protease site between GST and HED released both hIgG1Fc and HED proteins from the beads. Size exclusion chromatography was next employed to isolate the eluted proteins, and the retention volume of the main peak indicated that the HED-hIgG1Fc complex had an estimated MW of 49 kDa (Figure S1B). Further analysis by SDS-PAGE revealed two major bands: a 28 kDa band for hIgG1Fc and an 8 kDa band for HED (Figure S2B).

**Figure 2:**
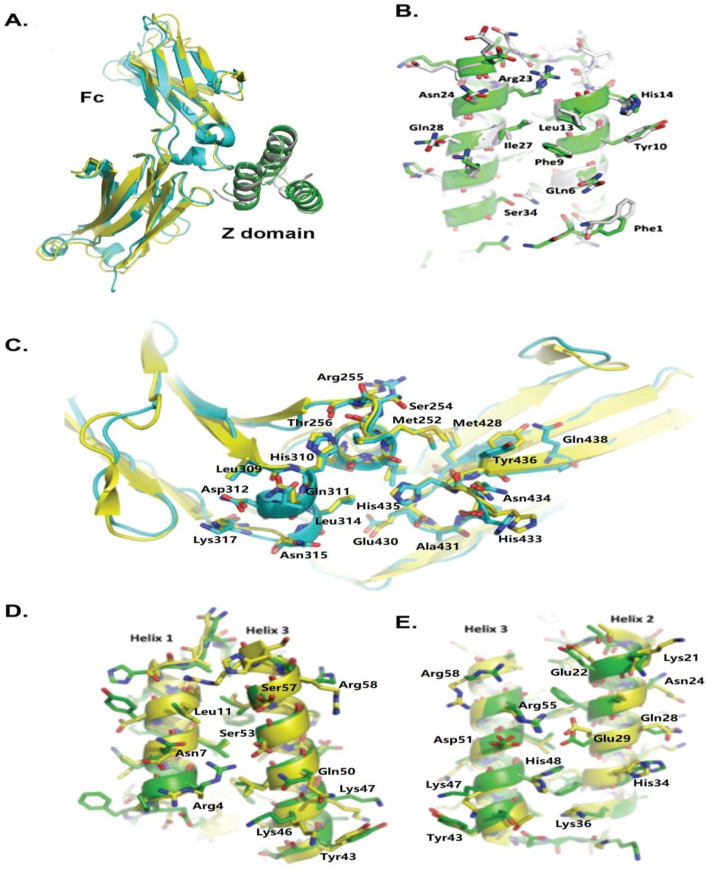
Crystal structure of HED domain. (**A**) Structure alignment of the complexes of the Fc fragment of IgG1with the HED domain and the B domain of SpA (PDB: 1FC2). The structure models are shown as illustrations: the B-domain of the SpA and HED domains are colored grey and green and the Fc fragment in PDB 1FC2 and in complex with HED are colored yellow and cyan. (**B**) Structure alignment of the HED domain and B-domain (1FC2). The residues in the B-domain and the HED domain involved in the interaction of the Fc fragments are shown as sticks on their illustrated structure. The carbon atoms in the B-domain and the HED domain are painted in grey and green. (**C**) Structure alignment of the Fc fragment in the complexes with the B-domain (PDB: 1FC2) and the HED domain. On their illustrated model, the residues in the Fc fragments involving the interaction with the B-domain or the HED domain are shown as sticks with their carbon atoms in yellow and cyan, respectively. (**D**,**E**). Structural alignment of the HED domain with the region of alpha-2-MRAP used for modeling. The models are shown as illustrations and the side chain atoms of HED, and alpha-2-MRAP are presented as sticks on illustrations and their carbon atoms are colored green and yellow, respectively.

The structural details of the interaction between HED and hIgG1Fc were resolved at the atomic level. The crystallization of the purified complex allowed for the solution and refinement of its structure to 2.7 angstroms. Table 1 displays data collection and structural refinement statistics. There is one hIgG1Fc chain and one HED chain in an asymmetric unit (Figure 2A). The cysteine residues at the N-terminus of the Fc fragment are flexible and not visible in the electronic map. The Fc was packed as an Fc dimer in the crystal, a feature seen in other crystal structures of Fc [3,22]. When superimposed on the Fc monomer of the new complex, the Fc’s alpha carbons from PDB 4WWI and IFC2 gave the mean RMSD values of 0.358 and 0.862 Å, respectively [3,22]. The alignment result shows that the angles of the complex between the CH2 domain and CH3 domain stretch less when compared with 1FC2, which may be due to the sugar component that normally exists in the two chains of the Fc fragment in 1FC2.

The HED domain folds into a three helical bundle in the complex crystal. The first and the second helixes are involved in the interaction with hIgGFc, while the third helix is tightly tethered to the hydrophobic core and resides on the opposite side of the Fc fragment. The structure involved in Fc-binding is conserved in the HED and the Z-domain from 1FC2 and 4WWI, for which the mean rmsds are 0.479, 0.420, and 0.487 angstroms, respectively. Similar to the complex structures of the Fc fragment with the SpA B-domain or the SpA C-domain [3,22], the Fc fragment of the human IgG1 interacts with HED by the elbow between the CH2 and CH3 domains.

The crystal structures of hIgG1Fc in complex with the HED and with the B-domain indicate that the Fc fragment interacts with the HED and B-domains in a similar manner (Figure 2A). As deduced by the structure alignment of Fc’s in PDB 1FC2, PDB 4WWI, and the HED complex, the Fc residues involved in interacting with the HED or the Z-domain of SpA are almost identical and most of the side chains adopt a similar conformation (Figure 2C). In the crystal structure of the HED complex with the Fc fragment, the main chain atoms of residues Q6, F9, Y10, L13, H14, R22, N23, I26, and Q27 of the HED domain, which were mutated from 2FCWF1, reside in identical positions of the Z-domain helixes and are involved in interactions with the Fc fragment (Figure S3). Notably, their side chains maintain similar orientations to the Fc fragment, and very few positional changes are observed for the distal atoms (Figure 2B).

S33 and Q37, which are distinct from residues of 2FCWF1, reside in the inserted loop between helix2 and helix3 of HED (Figure 1D and Figure S2). The crystal structure of the HED complex shows that the oxygen gamma of serine 33 is close to atoms of serine 135 in the Fc (Figure S3). In contrast to the long side chain of glutamic acid of 2FCWF1, the S33 avoids steric collisions with atoms of the Fc. In conclusion, the humanized Z-domain HED was presented with all the characteristics of the Z-domain, which enabled it to permit similar patterns of binding to the Fc segment of human IgG.

In addition to the Fc binding activity we created for the HED, we designed the new protein to retain specific three-dimensional structure features of the original human protein to reduce immunogenicity. For this reason, we only mutated the residues that participate in the interaction with the Fc fragment and residues that might introduce spatial effects. The crystal structure of HED could be perfectly aligned to the modeling region of hA2MRAP, with the mean rmsd of the superimposition at about 0.866 angstroms. The solvent-accessible surface of HED, except for the surface of helix1 and helix2, possess similar structural features as that of hA2MRAP (Figure 2D,E). The main chain atoms of the residues on the interface between helices 2 and 3 and helices 2 and 3 are the same as the main chain atoms of similar residues in hA2MRAP. The orientation of the side chain atoms changes very little, which is also seen in high-resolution structures of hA2MRAP.

### 2.3. IgG Fc Associated with HED Binds to the Fc Receptor on Effector Cells

In order to utilize host antibodies to recruit host cells that would kill specific target cells through the CDC and ADCC pathways, the HED must bind to the Fc and avoid interrupting binding of Fc to Fc receptors and C1q. We determined that the interaction of the HED with IgG Fc did not affect the binding of the Fc to the Fc receptor. This event is an essential step to induce ADCC, a biological activity we wished to maintain. The complex structures of human IgG1Fc with human Fc receptor revealed that Fc fragments, after interacting with the HED domain, have a normal binding pattern to Fc receptors [23,24]. An interaction model of the HED, Fc fragment, and Fc receptor was built based on our HED/Fc complex structure and the structure of the human IgG1Fc/human Fc gamma receptor IIIa complex (Figure S4A). The binding site of the Fc receptors resides within the hinge region of Fc, spatially distant from the elbow region of Fc that binds to the HED. We anticipated that the HED domain might be used as an Fc recruiter to induce ADCC, which can be tested experimentally.

Complement activation for the CDC pathway necessitates the critical step of C1q binding to the Fc domain of Ig molecules, namely, IgG or IgM complexed with Ags. The complex structure of human Fc fragments with complementary C1q molecules was used to generate a complex model of HED, Fc, and C1q [25]. The model shows that the interaction location between hIgG1Fc and the HED is far from the binding site of C1q on the Fc fragment (Figure S4B) and HED should not hinder the CDC activity mediated by the interaction of Fc with C1q. The structural analysis indicated that the ADCC and CDC events of this antibody-like molecule would occur in an unimpeded manner.

### 2.4. Fusion of scFv Fragments with HED to Produce an Antibody-like Structure That Potentiates ADCC and CDC by Recruiting hIgG1

We fused the scFv of 4D5 to the Z domain to generate 4D5scFv-ZZ [10]. In order to withstand degradation caused by hydroxylamine and cyanogen bromide [26], the Z-domain of SpA was modified from the B-domain and is well known to bind the Fc segment of IgG1 with an affinity of 10 nM [27]. In addition to capturing an IgG to guide effector cells expressing Fc receptors to breast cancer cells to produce ADCC, 4D5scFv-ZZ can bind to p185Her2/neu on the surface of breast cancer cell types. In vitro, 4D5scFv-ZZ produced strong ADCC activity of PBMCs against BT474 breast cancer cells in the presence of human IgG in the medium. T7-16 proliferation was reduced lytically in 5% mouse serum containing active complement components, indicating that 4D5scFv-ZZ exhibited significant complement-dependent cytotoxicity (CDC) [10].

**Figure 3:**
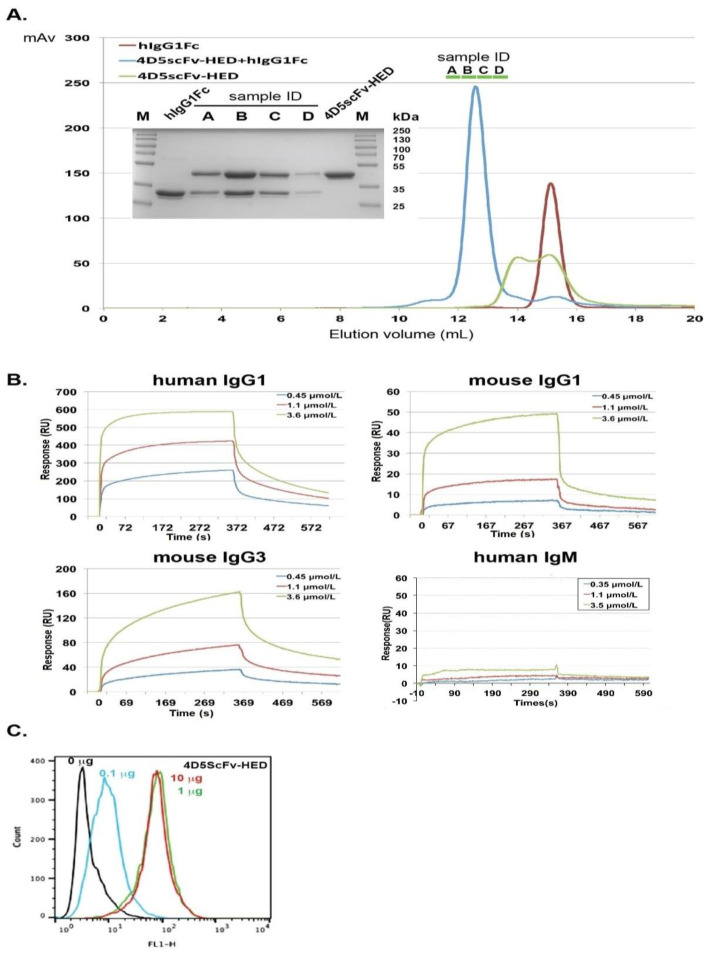
Binding activity of 4D5scFv-HED to intact IgGs or the Fc fragment of IgG1. (**A**) FPLC result shows the fusion protein of the HED domain and 4D5scFv could bind to the Fc fragment of IgG1 in solution. The curves of 4D5scFv-HED, Fc fragment, and their complex are colored in green, red, and blue, respectively. Insert: For each fraction of the peak, a 20 μL sample was loaded on a 12% SDS-PAGE, and coomassie brilliant blue staining solution was used to visualize the proteins. (**B**) Biacore assay to determine the binding of the fusion protein of HED and 4D5scFv to human IgG1, mouse IgG1, mouse IgG3, and human IgM. (**C**) FACS assay shows the binding of 4D5scFv-HED to T6-17 cells. After being washed by FACS buffer, cells were incubated without or with different concentrations of the fusion protein 4D5scFv-HED for 30 min followed by washing and incubation with FITC-labeled goat immunoglobulin, and then the cells were analyzed on a flow cytometer.

We prepared a plasmid to express the new 4D5scFv-HED by inserting the 4D5scFv and the HED into the vector pSectagA and expressed it in HEK-293T cells. The protein was purified by affinity chromatography and size exclusion chromatography. A human IgG1 Fc was also expressed and purified from HEK-293T cells. According to the filtration patterns on Superdex 200, the molecular weight of the Fc was determined to be 45 kDa and the molecular weights corresponding to two peaks of 4D5scFv-HED were 74 kDa and 46 kDa (Figure 3A). Based on these observations, the Fc can form dimers, and 4D5scFv-HED can be found in solution in both its dimer and monomer forms. A characteristic that has also been found in the crystal structure of the B-domain complex with IgM Fab is that the 4D5scFv-HED dimerizes via the surface of helix1 and helix2 of the HED domain [7].

The 4D5scFv-HED and IgG1Fc were mixed at a molar ratio of 1:1 and incubated at 4 °C for two hours. The mixture was loaded onto a Superdex 200 column and only one elution peak was detected, with the calculated molecular weight of 140 kDa. Subsequently, the collected fractions under the peak of the mixture of IgG1Fc and 4D5scFv-HED were examined using 12% SDS-PAGE (Figure 3A). The result confirmed that every fraction contains both Fc and 4D5scFv-HED, indicating that these two proteins form a complex in solution. The data indicate that 4D5scFv-HED and IgG1Fc can form tetramers in solution.

Biophysical binding of 4D5scFv-HED to Fc domains was characterized. We performed SPR studies using chips with several IgG species immobilized on them. As shown in Figure 3B, 4D5scFv-HED demonstrated good binding to human IgG1, mouse IgG3, and less to mouse IgG1 with KDs of 0.62 µM, 1.25 µM, and 3 µM, respectively, but not to human IgM, which possibly resulted from the surface change of helix2 and helix 3. In contrast, the fusion protein of 4D5scFv and the original 2FCWF1 fragment did not display binding activity to any of these immunoglobulins. This study confirms that 4D5scFv-HED binds to IgG Fc fragments (Figure S5).

### 2.5. Binding Potential of 4D5scFv-HED to Cell Surface p185Her2/neu

To confirm that the 4D5scFv fragment in the fusion protein can still bind to p185Her2/neu without being influenced by the HED binding to Fc, we performed FACS studies. T6-17 cells that express p185Her2/neu were incubated with different concentrations of 4D5scFv-HED, washed with buffer, and then incubated with FITC-labeled immunoglobulin to examine binding. The 4D5scFv-HED demonstrated a clear concentration-dependent binding activity (Figure 3C). The interaction of 4D5scFv with p185Her2/neu did not influence the binding of the HED surface to the Fc fragment of the FITC-labeled immunoglobulin.

### 2.6. New Species of HED Bodies Inhibits Tumor Cell Growth In Vitro and In Vivo

**Figure 4:**
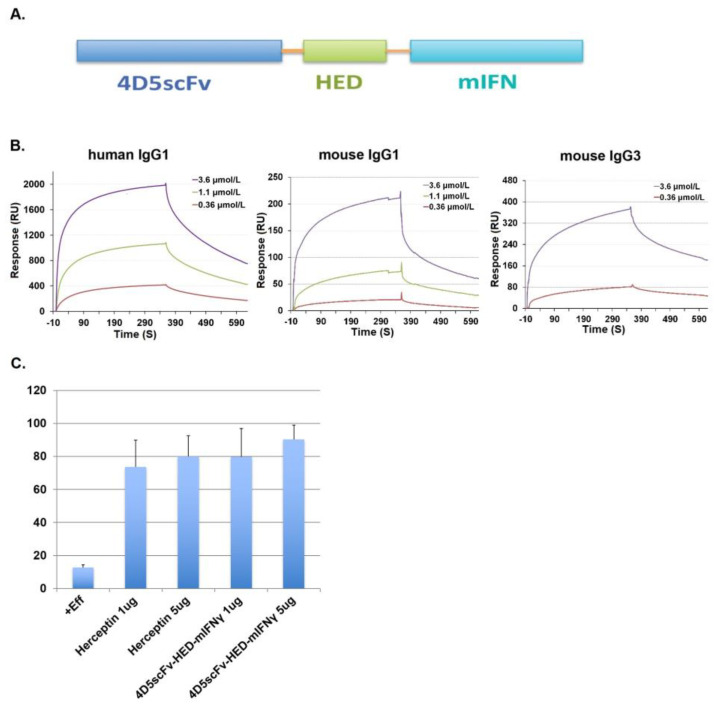
The function of 4D5scFv-HED-mIFN-γ. (**A**) 4D5scFv-HED-mIFN-γ was constructed by sequentially linking the single chain variable fragment of mAb4D5, the HED domain, and mouse interferon gamma together using a general linker of GGGGS. (**B**) Real-time surface plasmon resonance sensorgrams obtained after injection of the purified 4D5scFv-HED-mIFN-γ at different concentrations over sensor chip flow-cell surfaces containing human IgG1, mouse IgG1, and mouse IgG3. (**C**) ADCC activity of human NK cells against breast cancer BT474 cells was tested in the presence of Herceptin or 4D5scFv-HED-mIFN-γ at different concentrations. 4D5scFv-HED-mIFN-γ shows comparable ADCC activity with Herceptin.

IFN-γ is a cytokine that stimulates multiple elements of the immune system. Our recently reported in vivo research showed that IFN-γ may significantly slow tumor growth when given concurrently with or after monoclonal antibody administration [28]. Recently, our lab also developed a family of proteins called scFvZZ-IFN-fusion proteins, which exhibited greater in vivo efficacy than the anti-HER2 antibody 4D5 (Herceptin) [29]. A new species of HED body with a single chain Fv, followed by the HED domain and murine IFN-γ in a domain-ordered manner shown from the N-terminus to C-terminus in Figure 4A, was created. Similar to 4D5scFv-HED, this new HED body 4D5scFv-HED-mIFN-γ displayed binding activity to IgG Fc domains with KDs of 1.39 µM, 0.69 µM, and 0.30 µM to human IgG1Fc, mouse IgG1Fc, and mouse IgG3Fc, respectively (Figure 4B).

We determined whether the new scFv HED body species can lead to effector functions that are triggered by antibodies. Both ADCC and CDC activities were assessed using in vitro experiments. LDH release assay was used to measure the ADCC of 4D5scFv-HED-mIFN-γ against BT474 breast cancer cells [10]. Human NK cells were used as effector cells. Compared with Herceptin, comparable ADCC activity was observed with 1 µg/mL and 5 µg/mL of 4D5scFv-HED-mIFN-γ at an effector-to-target cell ratio of 2.5:1 and in the presence of 100 µg/mL human IgG (Figure 4C). This study confirmed that the 4D5ScFV-HED-IFN-γ induced ADCC against p185Her2/neu-expressing tumor cells in an IgG-dependent manner. Thus, the HED domain can be used as a fusion partner for scFv of antibodies to recruit Fcs to induce ADCC and CDC, which should enhance the therapeutic efficacy of current targeting monoclonal antibody therapies.

**Figure 5:**
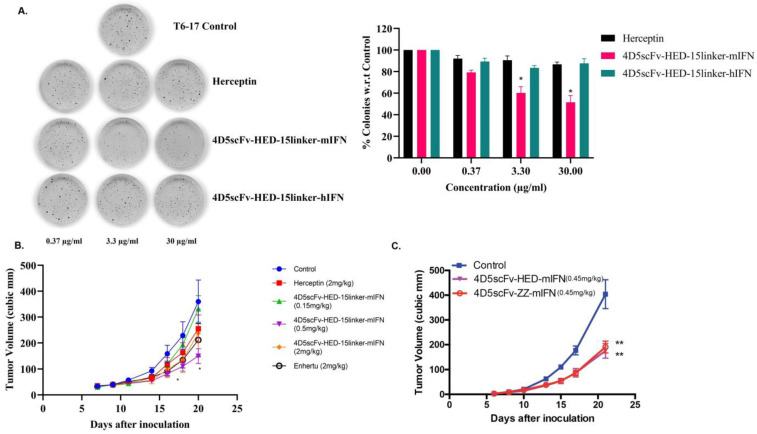
Functional characterization of HED engineered bodies in vitro and in vivo. (**A**) Colony formation assay against T6-17 cells using Herceptin, 4D5scFv-HED-15-linker-mIFN-γ and HED-15-linker-hIFN-γ, and Herceptin. Here, 4D5scFv-HED-15-linker-mIFN-γ shows dose-dependent cytotoxicity against T6-17 cells. (**B**) Targeted therapy of engineered antibodies (HED with 15 linker) against HER2 overexpressing murine CT26 cells. Enhertu is an antibody-drug conjugate (ADC) that targets HER2 and has been jointly developed and commercialized by AstraZeneca and Daiichi Sankyo. Mice were given treatment intraperitoneally every alternate day. The engineered protein 4D5-scFv-HED-15-linker-m-IFN significantly reduces tumor volumes depicting the efficacy of this powerful new platform. (**C**) T6-17 cells were injected into athymic nude mice, and the latter were treated with 4D5scFv-HED-mIFN-γ or 4D5scFv-ZZ-mIFN-γ were provided to mice (0.45 mg/kg/dose) intraperitoneally. Significant reductions in tumor volumes were observed. Error bars represent the SEM. (* *p* < 0.05, ** *p* < 0.01).

Studies were undertaken to examine if the HED domain influences anti-tumor activity. A linker of (GGGGS)3 was incorporated between the HED domain and functional fusion fragments to increase the pharmacological stability of these engineered proteins. We then tested the efficacy of engineered antibodies in vitro by soft agar assay at different doses using the T6-17 mouse cell line that over-expresses human p185Her2/neu [30]. We observed a dose-dependent inhibition of colony formation using 4D5scFv-HED-15-linker-mIFN-γ, but the cells were resistant towards 4D5scFv-HED-15-linker-hIFN-γ and Herceptin (Figure 5A), which may be a result of human interferon-γ having weak activity on the mouse IFN-γ receptor. Additionally, we evaluated the efficacy of 4D5scFv-HED-15-linker-mIFN-γ against HER2 overexpressing murine CT26 cells in athymic nude mice (Figure 5B). Herceptin was used as a control while Enhertu is an antibody-drug conjugate (ADC) that targets HER2 and has been jointly developed and commercialized by AstraZeneca and Daiichi Sankyo. The engineered protein 4D5-scFv-15-linker-HED-mIFN-γ significantly reduces tumor volumes clearly depicting the efficacy of this powerful new platform. In yet another tumor model using T6-17 cells, mice were administered 4D5scFv-ZZ-mIFN-γ, 4D5scFv-HED-mIFN-γ, or PBS. As shown in Figure 5C, both 4D5scFv-HED-mIFN-γ and 4D5scFv-ZZ-mIFN-γ showed similar activity and a significant tumor growth inhibition.

## 3. Discussion

In the past couple of years, the number of therapeutic protein products that can be used in clinical settings, such as monoclonal antibodies, cytokines, hormones, and engineered protein scaffolds, has grown substantially [31,32]. High specificity to target protein enables antibodies to distinguish tumor over heathy tissue, thus facilitating their successful use in the diagnosis and treatment of cancer [33]. However, poor penetration and heterogeneous distribution in solid tumor limit their clinical efficacy, leading to the failure to target a subpopulation of cancer cells that may subsequently contribute to tumor relapse [34]. To improve efficacy, antibody characteristics such as molecular size, valency, binding affinity, and pharmacokinetics have been modified for a variety of clinical applications [35]. The scFv (single chain fragment variable), which is smaller than the full length antibody but retains the antigen targeting capability, could function to block the action of biological molecules by binding either the ligand or receptor. ScFvs have also been employed to cancer diagnosis or treatment by fusing to a range of toxins such as cytotoxic protein, radionuclides, or drugs [36]. Without the Fc domain, scFv fails to induce the effector function such as antibody-dependent cell-mediated cytotoxicity and complement-dependent cytotoxicity. [37]. By combining the penetration and effector function of an antibody, our laboratory generated a small recombinant molecule termed Grababody 4D5scFv-ZZ by incorporating the Z-domain derived from Protein A into a 4D5scFv fusion protein, which could recruit circulating IgGs to regain ADCC and CDC activity targeting Her2-specific cancer cell lines. The Grababody also demonstrated much greater activity than 4D5scFv in the inhibition of tumor growth, which was comparable to the inhibition activity of the h4D5 antibody [10].

The Z-domain was derived from the B-domain of Staphylococcus aureus Protein A. It has been observed that during treatment with biologics, anti-therapeutic protein anti-bodies or anti-idiotypic antibodies can form, which can reduce the clinical effect of the drugs [38]. In order to reduce the potential immunogenicity of the Z-domain, we employed a three-dimensional structure of the Z-domain as a model to search the Protein Data Bank (PDB) for human proteins with similar structural folds. We chose proteins with stable structures, easy access to solvents, and few gaps in their primary sequences. Based on the structural alignment of the Z-domain and the modeling protein, we designed appropriate peptides to link the different parts and grafted Z-domain amino acid residues that interact with the human Fc fragment. The humanized engineered domain was termed HED and first expressed as a glutathione S transferase (GST) fusion protein with a TEV digestion site at the 5-prime terminus of HED. After co-transfection with hIgG1Fc into BL21(DE3), the co-expressed proteins could be co-purified by Glutathione Sepharose. The hIgG1Fc is bound to Glutathione Sepharose through interactions with the GST-HED fusion protein. TEV digestion on beads led to shedding of hIgG1Fc and HED together. When the hIgG1Fc and HED domains were purified using size exclusion columns, they were found in the same peak. This shows that the humanized Z-domain binds hIgG1Fc.

We crystallized the HED-hIgG1Fc complex and solved its structure to determine how the two molecules interact at the atomic level. The structure of the complex showed that HED folded in a way similar to the Z-domain and interacted with the Fc fragment in the same way as the Z-domains. Except for the Phe1 residue at the N-terminal, the HED domain maintained a conserved interface with Fc, displaying an unchanged frame for main chain atoms as well as an unaltered orientation for side chains. This structural feature is critical for interaction with the Fc fragment. Consistently, residues of the Fc region involved in the interaction with the HED or Z-domain maintained the main chain structure and underwent little change for the side chain orientation. Moreover, other HED surfaces not participating in the interaction with Fc also maintained the homologous structure of the hA2MRAP fragments. This was shown in the three-structure alignment typically represented by the residues on the surface of helix2 and helix3. Residues that underwent change resided in the linker between helix2 and helix3, an area that does not exist in the structure of the 2fcw fragment. Therefore, the HED had the same structure as the original human protein it was generated from, except for the residues that formed the interface with the Fc fragment.

SpA Z proteins were found to bind to the Fab of IgM. However, the structure of HED indicated that the residues on the surface of helix2 and helix3 were different from those in the Z-domain that contribute to its interaction with the Fab fragment of human IgM. Consistently, the SPR assay showed 4D5scFv-HED and, unlike the Z-domain, could not bind to human IgM. By binding to VH3 clan membrane-bound immunoglobulins, the Z-domain could induce expansion of B cells. Therefore, the humanized HED domain should have reduced immunogenicity as it lacks VH3 affinity.

Cytokines are potent immune-modulating proteins that are used in cancer treatment [39]. IL-2 is one of the FDA-approved immunocytokines for metastatic melanoma and renal cell carcinoma [40]. IFN-α was FDA approved to treat hematological malignancies and melanoma at high dose [41]. Cytokines combined with antibodies were used in clinical trials to enhance the systemic and intratumor immune responses such as IL2 with nivolumab [42] and IFN-γ with Bevacizumab [43]. We reported that a combination of IFN-γ with anti-HER2 antibody synergistically reduced tumor growth in an in vivo implanted mammary tumor model [28]. Clinical application of cytokines is still limited due to their short half-life in vivo, severe toxicity at therapeutic doses, and overall lack of efficacy. Antibody-cytokine fusion proteins, such as anti-CD20/IFN-α, SumIL2/anti-EGFR, an-ti-PD1/IL-21, anti-EGFR/IL-21, arose to target these molecules in the tumor environment and demonstrated an improvement in either increased antitumor efficacy or reduced toxicity [44]. Our engineered protein by coupling Grababody with IFN-γ was also proved to change the tumor microenvironment to support immune activity against tumor [29]. Here, the humanized Grababody 4D5scFv-HED was further fused with IFN-γ to generate a new species of antibody-like molecules, which we term HED bodies. In the HED body, the binding activity of the HED domain to Fc fragments is maintained in the SPR assay. In the presence of human IgG1, the HED body showed strong ADCC activity by recruiting human NK cells to kill target cells. In the colony formation assay, the HED body with mouse IFN-γ showed clear inhibition on T6-17 cells, which have mouse IFN-γ receptors. With ADCC and CDC activity, HED bodies that contain either the humanized HED domain or the bacterial Z domain had comparable in vivo activity in the inhibition of HER2-transformed tumor growth in rodents. This result provides a new foundation to generate therapeutic fusion proteins with other therapeutic cytokines.

In conclusion, based on the three-dimensional structure of the Z-domain of SpA, we constructed a novel human-engineered domain (HED) structure that was derived from the human Alpha-2-macroglobulin receptor-associated protein. The novel HED domain with Fc binding ability could serve as a promising module in a fusion protein scaffold for developing therapeutic interventions against infectious and malignant diseases.

## 4. Materials and Methods

### 4.1. Model Design

In order to create a humanized version of the Z-domain of Staphylococcus aureus protein A (SpA), the crystal structure of the D-domain of SpA in complex with the Fab fragment of human IgM antibody (PDB code: 1DEE) was used as a model to search for a similar three-dimensional structure in the Protein Data Bank on the DALI webserver [16]. From the Dali output, we selected human proteins with low RMSD and excluded those in which the similar structure parts are not solvent accessible. Then, we superimposed them on the crystal structure of the Z-domain in Pymol and chose the smallest corresponding regions of the Z-domain as initial models. Then, we superimposed the complex structure of the SpA B-domain to IgG1Fc (PDB code: 1FC2) on initial models and the residues of this Z-domain involving the interaction with IgG1Fc were identified as the mutated target residues from the overlapped or spatially close residues of initial models. Finally, the cleaved chains of the models were linked by a small linker similar to that in the original structure of the helices. After determining the modeling regions and the specific mutation residues, we obtained the amino acid sequences of the humanized Z-domain (HED).

### 4.2. Protein Expression and Purification

The cDNA for HED was synthesized and subcloned into vector PTG (which was modified from pET28a by insertion of the genes of GST and TEV Protease recognition site between Nde I and BamH I) to generate a GST fusion protein, GST-HED. The plasmid pET21a-hIgG1Fc, containing the human Fc fragment of human IgG1, was constructed from the vector pET21a. Subsequently, the two plasmids, PTG-GST-HED and pET21a-hIgG1Fc, were co-transfected to Escherichia coli Blx using 100 μg/mL ampicillin and 50 μg/mL kanamycin as selection antibiotics. A single colony was inoculated into 25 mL of Luria broth media with 100 μg/mL ampicillin and 50 μg/mL kanamycin. The starter culture was incubated and shaken at 220 rpm at 37 °C overnight, whereupon it was used to inoculate 0.75 L cultures that were allowed to grow at 37 °C to an OD of about 0.8–1.0 at 600 nm. Then, the culture was cooled down to 16 °C and isopropyl-beta-d-thiogalactopyranoside (IPTG) was added to a final concentration of 0.5 mM, and the cultures were incubated for an additional 20 h. The cells were harvested by centrifugation and resuspended in 80 mL lysis buffer containing 20 mM phosphate buffer pH 8.0, 500 mM sodium chloride, 1 mM PMSF, 1 mM DTT, and a protease inhibitor cocktail. The cells were lysed by sonication and the supernatant was separated from the pellet by centrifugation at 20,000× *g* for 30 min at 4 °C. Then, the supernatant solution was incubated with 2 mL Glutathione-Sepharose beads overnight at 4 °C in lysis buffer and the beads were washed 3 times using 10 mL lysis buffer and 2 times using 10 mL TEV protease digestion buffer consisting of 50 mM Tris pH 8.0, 100 mM sodium chloride, 1 mM DTT, and 0.5 mM EDTA. The mixture of beads and proteins was split into about twenty aliquots. For each aliquot, 14 mL of digestion buffer and 0.5 mL 1 mg/mL TEV was added for digestion on beads. After 8 h of incubation at room temperature, 40 μL mixture was set aside for SDS-PAGE assay and the other solution containing the complex of HED and hIgG1Fc was separated from the beads. The complex solution was pooled together and concentrated for further purification using size exclusion chromatography (Superdex 200) in digestion buffer on an Akta FPLC purifier system. The fractions containing the complex of hIgGFc and HED were collected, concentrated, and dialyzed against the buffer of 20 mM tris pH 8.0, 100 mM sodium chloride, and 1 mM DTT. Subsequently, the complex was concentrated to 18 mg/mL for further research.

At the same time the DNAs of hIgG1Fc and novel Grababody 4D5scFv-HED with His-tag (single chain variable fragment fusion of 4D5 with humanized Z-domain) were cloned separately into pSectagA expression vectors. Then, plasmids were transfected to human embryonic kidney (HEK) 293T cells by lipofectamine 2000 using the standard transfection procedure. The target proteins were purified from the medium using Nickel-Sepharose beads after concentration. The proteins were further purified on an AKTA FPLC using Superdex 200 in PBS and stored at −80 °C. In order to test the binding of the humanized Z-domain to the human Fc fragment in solution, hIgG1Fc and 4D5scFv-HED of equal molarity were incubated together for 2 h at 4 °C and loaded on Superdex 200 of the AKTA FPLC for a binding assay. For each fraction of the peak, a 20 μL sample was loaded on a 12% SDS-PAGE and Coomassie brilliant blue staining solution was used to visualize the proteins.

### 4.3. Crystallization, Data Collection, and Structure Determination

The complex of hIgG1Fc and HED was diluted to about 9 mg/mL in buffer (20 mM tris pH 8.0, 100 mM sodium chloride and 1 mM DTT) and mixed in a 1:1 ratio with crystallization solution (50 mM cesium chloride, 0.1 M MES monohydrate, pH 6.5, 30% *v/v* Jeffamine M-600) at 4 °C. Diffracting crystals were formed by hanging-drop vapor diffusion after several weeks and were then directly flash-cooled in liquid nitrogen. Diffraction data were collected at 100 k on beamline A1 at MacCHESS. The diffraction data were reduced using the HKL package, and the statistics of data collection and processing are summarized in Table 1. The structure of the hIgG1Fc-HED complex was solved using the molecular replacement program Molrep using the complex structure of hIgG1Fc and the B-domain of SpA (PDB code: 1FC2) as a search model. The structural model was built using Coot and was refined using Refmac5 and PHENIX. The refinement statistics are provided in Table 1. All the structural figures were prepared in Pymol (http://www.delanoscientific.com/ (accessed on 5 February 2023)).

### 4.4. Surface Plasmon Resonance (Biacore) Studies and FACS

To characterize the binding of HED as a fusion protein with 4D5scFv to immunoglobulins, we carried out surface plasmon resonance-based experiments using the biosensor instrument Biacore 3000 at 25 °C. Immobilization of mouse IgG1, human IgG1, mouse IgG3, and human IgM and the generation of a reference surface were conducted following the standard amine coupling procedure according to the manufacturer’s instructions. Binding of 4D5scFv-HED to these immunoglobulins on the chip was monitored in real time as a series of sensorgrams. Kinetics constants were estimated by global fitting analysis of the sensorgram curves to the Langmurian interaction model. The Kon and Koff were determined from the association and dissociation part of the sensorgram, respectively. The equilibrium dissociation constant (KD) was calculated as the Koff/Kon ratio.

T6-17 cells express HER2 and can be recognized by 4D5scFv. The FACS buffer (cold PBS containing 0.05% bovine serum albumin) washed cells were incubated without or with the fusion protein 4D5scFv-ZZ or 4D5scFv-HED in a volume of 0.2 mL FACS buffer for about 30 min on ice. After being washed, the cells were incubated with FITC-labeled goat immunoglobulin. Then, the mean fluorescence intensity (MFI) of each sample was recorded after FACS analysis of the stained cells.

### 4.5. ADCC Assay

Cytotoxicity was determined with the CytoTox 96 Cytotoxicity Assay Kit (Promega, Madison, WI, USA) using healthy donor human NK cells obtained from the Human Immunology Core at the University of Pennsylvania as effector cells at an E:T ratio of 2.5:1. Briefly, target cells (1 × 104/well) were seeded into flat-bottom 96-well plates (Nunc) the night before the experiment. Proteins were incubated with 20 μg of human IgG1 for 30 min at 4 °C in PBS and then added into wells. NK cells were then added (2.5 × 104/well) in a total volume of 100 μL for 4 h at 37 °C with 5% CO_2_. After incubation, the supernatants were collected and subjected to LDH measurement according to the kit’s manufacturer’s instructions. LDH release (%) was calculated as ([A]sample − [A]minimum)**/**([A]max − [A]minimum) × 100%, where [A]max is the absorbance value of a positive control (Triton X-100) in which complete target cell lysis occurred and [A] minimum is the negative control (without effector cells).

### 4.6. Soft Agar Assay

Carcinogenesis is characterized by anchorage-independent growth, which is the capacity of altered cells to expand without support from a solid surface. One of the most rigorous assays for cell malignancy is the soft agar colony formation assay, which is a well-established technique for assessing this capacity in vitro. Here, HBSS was used to make 2.5% Noble agar, which was then autoclaved before use. Later, 10% FBS in 1X DMEM (without Phenol Red; 21063-029 Thermo Fisher Scientific, Waltham, MA, USA; CC cat. 1234) was used. The bottom layer agar was used at 0.5%. T6-17 cells were trypsinized and seeded at a density of 10,000 cells/plate and later kept at 37 °C in an incubator. Cells were later treated with media, Herceptin, 4D5scFv-HED-15-linker-mIFN-γ, and 4D5scFv-HED-15-linker-hIFN-γ at different doses every 3 days. Plates were stained with p-INT violet after three weeks and then photographed. The percentages of the colonies were later analyzed using ImageJ software 1.54a.

### 4.7. In Vivo Xenograft Model

Athymic nude mice (Jackson labs) were injected with 0.5 x106 T6-17 cells into the right flank. After a period of about 1 week, mice were randomized and injected with PBS, 4D5scFv-ZZ-mIFN-γ, and 4D5scFv-HED-mIFN-γ, respectively, every alternate day. In another set of experiments, mice were injected with 1 x106 CT26 Her2 cells. Here, the mice received PBS, Herceptin, 4D5scFv-HED-15-linker-mIFN-γ, and Enhertu as described above. Tumor volumes were measured using Vernier calipers every alternate day. Mice were sacrificed after 3 weeks. Tumor volumes were then calculated and plotted using GraphPad prism software version 6.0.

### 4.8. Study Approval

Mice were housed and procedures carried out in facilities provided by the University of Pennsylvania which is AAALAC accredited. Procedures were approved by the Institutional Animal Care and Use Committee (IACUC) of the University of Pennsylvania.

## Figures and Tables

**Table 1:** Data collection and Structure refinement statistics.

Parameters	Values
**Data Collection**	
Space group	P31 2 1
Cell dimensions	
a, b, c (Å)	100.16, 100.16, 84.57
α, β, γ (°)	90, 90, 120
Resolution (Å)	20–2.7 (2.75–2.70) ^a^
R_merge_	0.072 (0.956)
Average I/σI	52.2 (3.0)
Completeness (%)	99.9 (100)
**Structure Refinement**	
Resolution (Å)	20–2.70
Unique reflections	13,703
Unique reflections for R_free_	724
No. of protein atoms/solvent	2199/31
R_crystallography_ (%)	19.13 (30.5)
R_free_ (%)	24.25 (43.0)
R.M.S deviations	
Bond length (Å)/angle (°)	0.008/1.207
Average B factor (Å^2^)	83.50
Ramachandran plot	
Most favored (%)	93
Allowed (%)	6.7

^a^ represents the value for the last resolution shell.

## Data Availability

The data presented in this study are available on request from the corresponding author.

## Supplementary Materials

The following supporting information can be downloaded at: https://www.mdpi.com/article/10.3390/ijms24076477/s1.
