# Supplementary material for: HED, a Human-Engineered Domain, Confers a Unique Fc-Binding Activity to Produce a New Class of Humanized Antibody-like Molecules
Source: Int J Mol Sci. 2023 Mar 30;24(7):6477. doi: 10.3390/ijms24076477 (PMC10094569; doi:10.3390/ijms24076477)
Supplement: Supplementary file 1 [file ijms-24-06477-s001.zip › ijms-2193155-supplementary.pdf]

Supplement Information

A. Prediction of immunogenicity by the T Cell Epitope Prediction Tool from the Immune Epitope Database (IEDB)

We have studied the immunogenicity of the SpA_B domain and HED using the immunogenicity model created by Calis et al. (Calis JJ, Maybeno M, Greenbaum JA, Weiskopf D, De Silva AD, Sette A, et al. Properties of MHC Class I Presented Peptides That Enhance Immunogenicity. PloS Comput Biol (2013) 9:1003266. doi: 10.1371/journal.pcbi.1003266). This model, which is available for iedb (http://tools.iedb.org) , scores peptides based on the position of an amino acid in a 9-mer peptide and the preference of such an amino acid by immunogenic peptides. With this model, 66% of the immunogenic peptide-MHC-I complexes (pMHCs) got a positive score, compared to 44% of the non-immunogenic pMHCs.

The SpA_B domain has 44 9-mer peptides, with 21 peptides (46%) showing a positive score in the immunogenicity model (Figure S1A). In comparison, HED domain has 52 9-mer peptides, with 20 peptides (38%) showing a positive score in the immunogenicity model. Therefore, the model indicates that HED has less immunogenic MHC-1 peptides.

As predicted by this model, both the SpA_B domain and HED are not very immunogenic. Although two peptides SpA_B have slightly higher scores (0.32699 and 0.31005) than any other peptides in SpA_B and any peptides in HED, the average scores of all peptides in both proteins are less than 0 (Figure SX2).

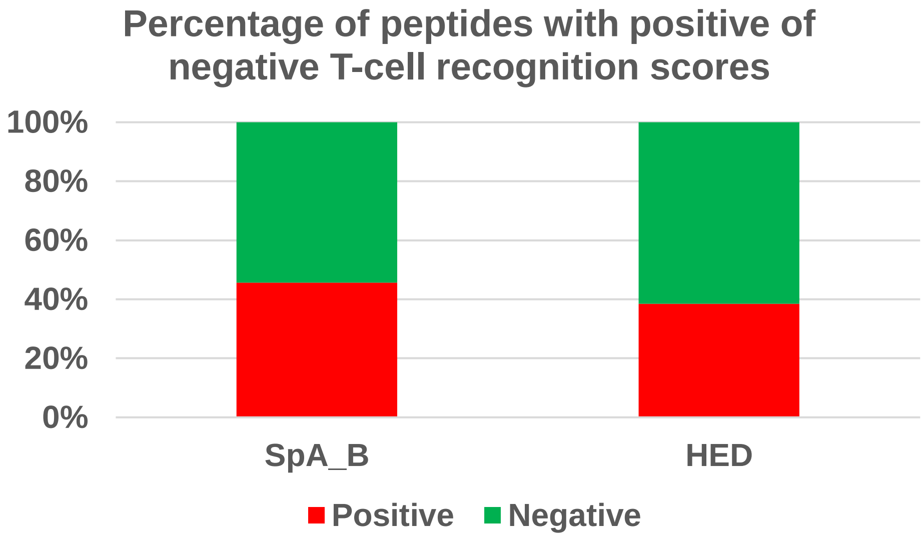

Figure S1A. Percentage of 9-mer peptides in SpA_B and HED with positive or negative T-cell recognition scores.

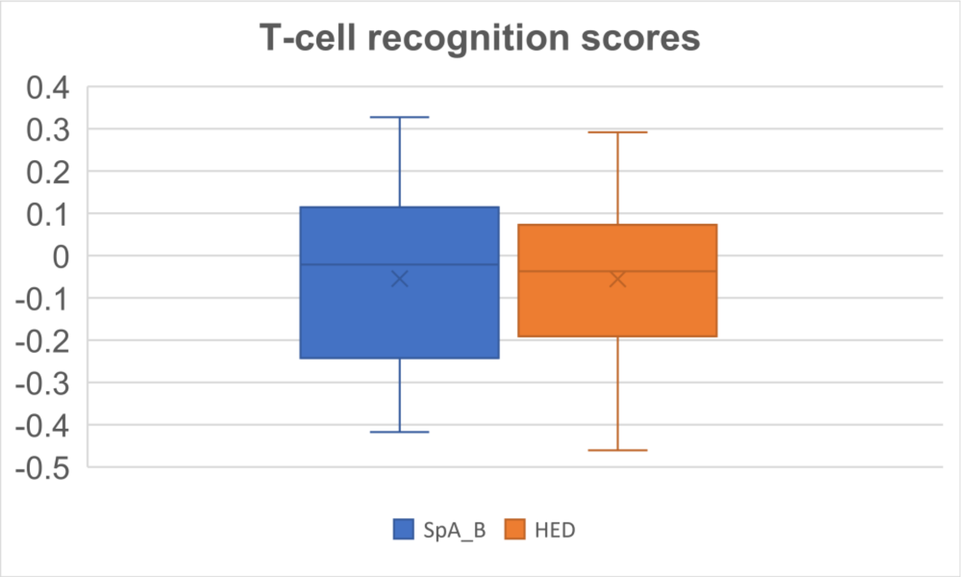

Figure S1B. Distribution of T-cell recognition scores of 9-mer peptides in SpA_B and HED.

B. MHC-II analysis

Analysis of MHC II binding predictions was performed using the IEDB analysis resource Consensus tool with the 7-allele HLA reference set (Wang P, Sidney J, Dow C, Mothé B, Sette A, Peters B. 2008. A systematic assessment of MHC class II peptide binding predictions and evaluation of a consensus approach. PLoS Comput Biol. 4(4):e1000048 and Wang P, Sidney J, Kim Y, Sette A, Lund O, Nielsen M, Peters B. 2010. Peptide binding predictions for HLA DR, DP and DQ molecules. BMC Bioinformatics. 11:568). Potential high and medium binding sites were identified in both the SpA-B domain and the HED domain. Although these are only in silico predictions and need validation with wet experiments, it reiterates our expectation that the HED may require further improvement to lower immunogenicity, even it is derived from a human protein.

C.

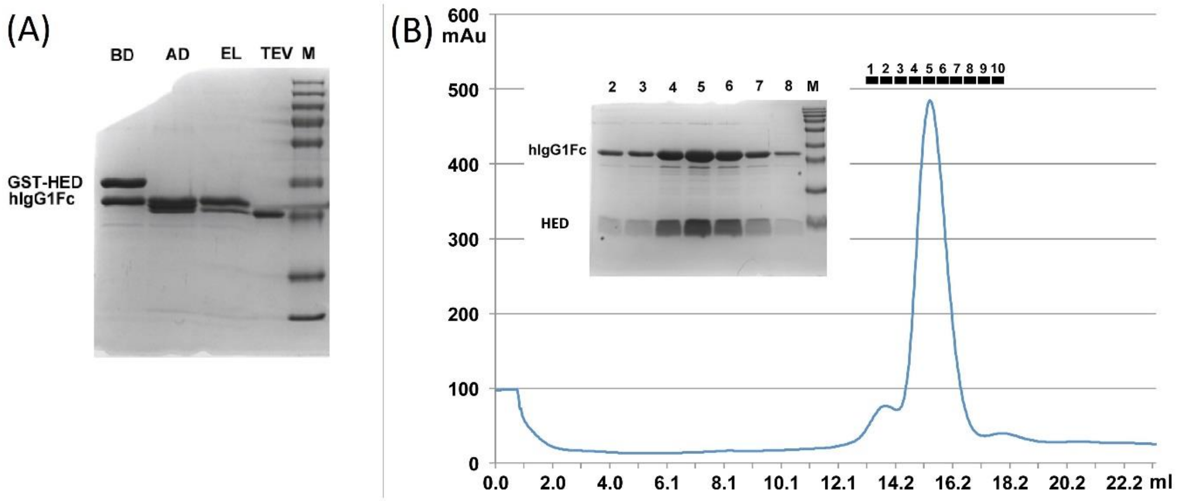

Figure S2. Co-purification of HED and Fc fragment of human IgG1(hIgG1Fc). GST-HED and hIgG1Fc were co-expressed in Bl21(DE3) and extracted from the bacteria. Then the supernatant was mixed with Glutathione-Sepharose beads overnight and washed by binding buffer and TEV (tobacco etch virus) protease digestion buffer, then digested by TEV protease for 8 hours at room temperature. Then the sample from binding bead before digestion (BD), the sample from binding bead after digestion (AD), the sample from eluted in digestion buffer (EL) and the TEV protease were load on 12% SDS-PAGE with PageRuler™ Plus Prestained Protein Ladder (M) from Invitrogen (shown in A). The digested off protein solution was pooled together, concentrated and loaded on Superdex 200 10/300 GL column for further purification (shown in B). The fractions were collected and loaded on 18% SDS-PAGE (shown in the insert of B).

D.

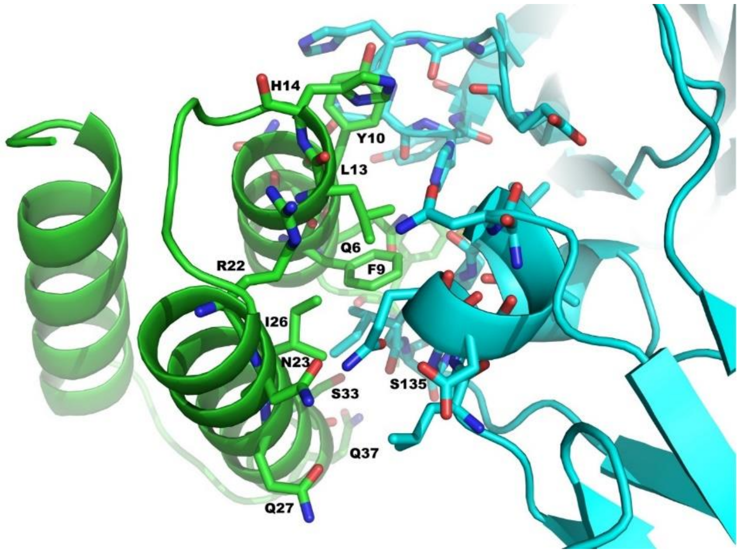

Figure S3. Interaction surfaces of HED and IgG1Fc. In the interfaces of HED and IgG1Fc, residues of HED domain mutated from 2FCWF1 are presented as sticks on the cartoon model with carbon atoms in green. The residues of IgG1Fc involving in interaction with HED are shown as sticks on the cartoon model in cyan.

E.

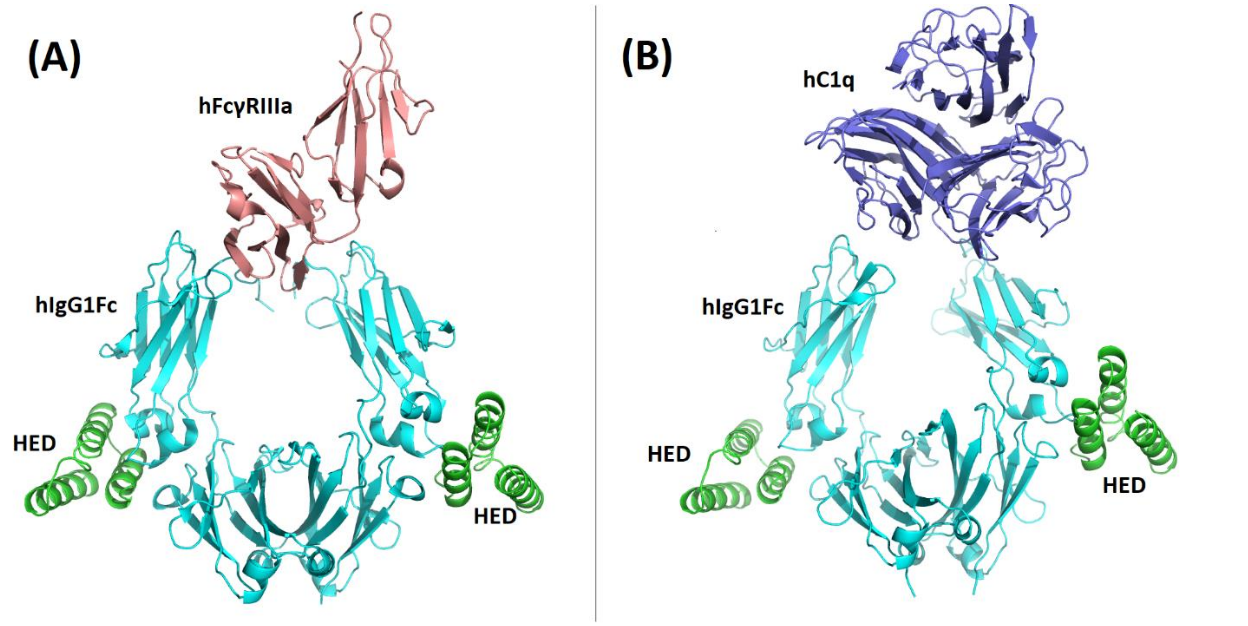

Figure S4. Interaction model of the complex of human Fc and HED with human Fcγ receptor or human C1q molecules. By superposing the Fc fragment of HED and hIgG1Fc complex to the Fc fragment of its complex with human Fc receptor IIIa, the interaction model of HED, Fc and Fc receptor was generated (A). Similar superposition was performed to generate the model of HED, Fc and C1q molecules (B). HED domain, human IgG1Fc, human Fc gamma receptor IIIa and C1q molecules are presented as cartoon and painted in green, cyan, pink and blue respectively.

F.

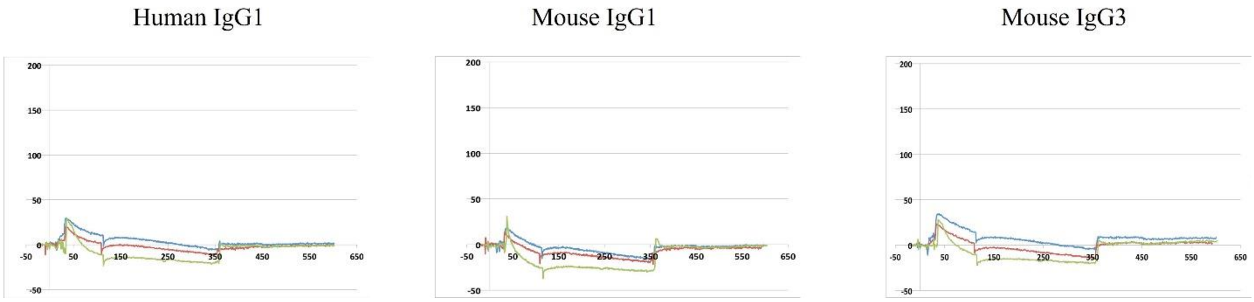

Figure S5. Biacore assay to determine the binding activity of the fusion protein of 2FCWF1 with 4D5ScFv to Human IgG1, mouse IgG1 and mouse IgG3.
